# Pilot Study of Polymerization Dynamics in nMAG Dose Gel

**DOI:** 10.3390/gels8050288

**Published:** 2022-05-06

**Authors:** Mantvydas Merkis, Benas Gabrielis Urbonavicius, Diana Adliene, Jurgita Laurikaitiene, Judita Puiso

**Affiliations:** Physics Department, Kaunas University of Technology, 44249 Kaunas, Lithuania; benas.urbonavicius@ktu.lt (B.G.U.); jurgita.laurikaitiene@ktu.lt (J.L.); judita.puiso@ktu.lt (J.P.)

**Keywords:** X-ray irradiation, gel dosimetry, polymerization dynamics, QA, photospectrometry

## Abstract

The essential component of modern radiation therapy is the application of steep dose gradients during patient treatment in order to maximize the radiation dose to the target volume and protect neighboring heathy tissues. However, volumetric dose distribution in an irradiated target is still a bottleneck of dose verification in modern radiotherapy. Dose gels are almost the only known dosimetry tool which allows for the evaluation of dose distribution in the irradiated volume due to gel’s polymerization upon irradiation. The accuracy of dose gel dosimetry has its own obstacle, which is related to the continuation of the gel’s polymerization after the radiation treatment procedure is finished. In this article, a method to monitor the polymerization dynamics of dose gels in real-time is proposed using a modified optical spectrometry system. Using the proposed method, the changes of the optical characteristics of irradiated nMAG dose gels in situ were assessed. The investigation revealed that the detectable polymerization in dose gel proceeds up to 6 h after irradiation. This time is significantly shorter compared with a commonly recommended 24 h waiting time allocated for polymer gel to settle. It was also found that dose rate significantly influences the temporal response of the nMAG dosimeter. By increasing the irradiation dose rate by a factor of 2, the time needed for the polymerization process to settle was increased by 22%. Identification of the gel’s post-irradiation polymerization time interval and its dependence on irradiation parameters will contribute to more accurate dose verification using dose gel dosimetry.

## 1. Introduction

The main goal of modern radiation therapy is the precise delivery of a radiation dose to the cancerous tissue while minimally affecting surrounding healthy tissues. Modeling is most commonly used for the theoretical evaluation of interaction processes and the planning of procedures, but experimental evaluation with the help of dosimeters is necessary to control possible negative effects [1]. Particularly large deviations from intended dose distribution in the target may occur when steep dose gradients, which are an integral part of modern radiotherapy, are used for a patient’s treatment [2]. This can lead to the risk of healthy tissue irradiation with high radiation doses [3].

In order to evaluate doses delivered to the target, radiation dosimeters are used. Among the wide variety of dosimeters (ionization chambers, films/self-films, optically stimulated (OSL), thermoluminescent (TLD), chemical, semiconductor dosimeters), dose gels are the right choice for volumetric dose distribution measurements used for the treatment dose verification [4]. Dose gels work within a wide range of energies and allow a volumetric dose distribution with a practically unlimited spatial resolution to be acquired depending on the dose evaluation (readout) technique and method [5]. Moreover, dosimetric gels are almost tissue-equivalent in terms of ionizing radiation absorption [6].

The working principle of dose gels is based on radiation-induced polymerization reactions [7]. The dose gel generally consists of a gelatin matrix, water, and monomers (M_n_) which react with free radicals (R**˙**) produced during the radiolysis of water. Radiation-induced changes of dose gel structures are described below [8]:

H_2_O → 2R (1)

R**˙** + M_n_ → M**˙**_n_ (2)

In the initiation phase, created polymer radicals (M**˙**_n_) react with more monomers and polymers (M_m_), resulting in the formation of long polymer chains in the gel (M**˙**_(n+m)_) [9]:

M**˙**_n_ + M_m_ → M**˙**_(n+m)_ (3)

Polymerization can be terminated by a combination of two radicals or by disproportionation–hydrogen atom transfer from one polymer radical to another, creating two non-radicals [10,11].

M**˙**_n_ + M**˙**_m_ → M**˙**_(n+m)_ (4)

M**˙**_n_ + R**˙** → M_n_ (5)

M**˙**_n_ + M**˙**_m_ → M_n_ + M_m_ (6)

M**˙**_n_ + M_m_ → M_n_ + M**˙**_m_ (7)

The degree of polymerization depends on the dose absorbed in the gel, which is also responsible for structural changes and variations of the physical and chemical properties of the irradiated gels [12]. Polymerization-induced changes can be registered using various techniques: magnetic resonance imaging (MRI) imaging, X-ray (CT) and optical computed tomography (OCT), optical scanning, sonography, and other methods [13].

Various formulations of dose gels exist: nMAG, nPAG, NIPAM, NIBMA, VIPET, etc. [6,14,15]. One of the highest dose sensitivities among gel dosimeters is achieved with crosslinker-free nMAG dosimetric gel, where methacrylic acid is used as a monomer [16]. The acronym nMAG describes the type and the main components of the dosimetric gel (normoxic methacrylic acid in gelatin dosimeter). The dose sensitivity of the dose gel can be enhanced by adding metal nanoparticles (mainly Ag or Au) [17,18].

It is important to mention that the 3D dose distribution observed in irradiated dose gels can be used as an experimental tool for the verification of the dose distribution obtained using a treatment planning system. Therefore, it is particularly important to ensure a well-defined, stable in time and space dose response [19].

Two parallel processes influence the dose response of the gel dosimeter after the irradiation: gelation and post-irradiation polymerization [20]. Gelation is the process when the transition the from liquid to semi-liquid phase occurs as a consequence of the formation of macromolecular structures from a branched polymer structure. The gelation process is very rapid when the temperature of the solution drops below 35 °C, but evolution is much slower in later phases. This process can continue for up to 30 days and contributes to the reduction of dose gel sensitivity [21]. Post-irradiation polymerization reactions progress up to 10 h after the irradiation [10,22] due to the presence of long-term radicals. In order to control the influence of temporal dose response variations on the final result, the reading of the dosimetric information is usually performed at least 24 h after irradiation, assuming that this time period is sufficient enough to achieve the thermodynamic equilibrium of the system [23]. This assumption was taken due to the lack of information regarding the time-dependent post-irradiation polymerization of dose gels, which is limited by the complexity of the required measurement method and the necessity of analyzing a large amount of generated data.

In this paper, a unique measurement system and methodology is proposed that includes the development of the hardware which allows for in situ dynamic measurements of the optical characteristics of irradiated gels during and after the irradiation procedure. The developed system can collect data for a practically unlimited period of time. Setting 24 h as the measurement time limit was based on the recommendations of other authors [22].

It is expected that the detailed measurement of polymerization dynamics using the described methodology will allow for the reduction of a dosimeter’s storage time before readout procedures, thus making the dose evaluation procedure much faster.

The exploration of the proposed measurement methodology allowed for the continuous investigation of polymerization dynamics in nMAG and silver nanoparticle-enriched Ag-nMAG gels during and after the irradiation procedure and evaluation of the impact of additives on the temporal dose response of gel dosimeters.

## 2. Results and Discussions

**Figure 1:**
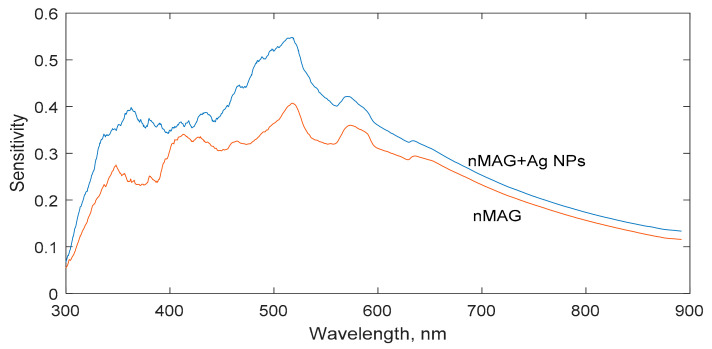
Variations of dose sensitivity of nMAG and nanoparticle-enriched nMAG dose gels.

For the evaluation of dynamic characteristics, the wavelength was identified, from which the largest change of the optical characteristics over time was observed. Using developed MATLAB code, the slopes of the dose-absorbance calibration curves were acquired at every available wavelength. The slope of the dose-optical absorbance calibration curve was considered as a measure of the dose sensitivity of the detector. Dose sensitivities of nMAG and silver nanoparticle-enriched nMAG dose gels are shown in a Figure 1. It should be noted that the nanoparticle-enriched dosimeters indicated a higher absorption ability compared with the pure nMAG gel dosimeters, but the signal to noise level was also higher.

The dose sensitivity curves of both dose gel formulations were similar. Significant variations of dose sensitivity were found at lower wavelengths, at which highly noisy optical absorption spectra were registered. Due to radiation-induced polymerization and the formation of the polymerized segments in the irradiated gels along with the presence of nanoparticles in the gels, the signal to noise ratio in a short-wave interval of the UV-Vis absorbance spectra decreased dramatically after irradiation, thus leading to high measurement uncertainties. In order to reduce the measurement uncertainties influenced by the noise level, all evaluations of the optical parameters of the irradiated dose gels were performed at the wavelength of 600 nm, where significantly less noisy signals were registered. It should be noted that the dose sensitivity of the gels was derived from the UV-Vis absorbance spectra.

**Figure 2:**
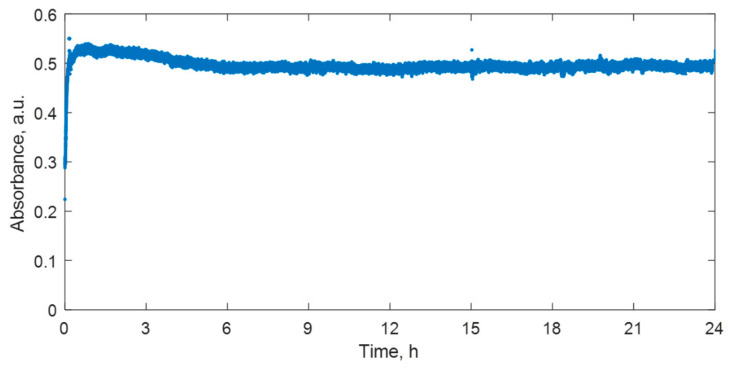
Variations of optical properties over 24 h in irradiated nMAG dose gel.

The polymerization dynamics in nMAG dose gel irradiated with a 1 Gy dose was followed over 24 h (Figure 2). The process was divided into two periods: the irradiation period (dose rate 300/cGy, 1 Gy dose was achieved in 20 s) and post-irradiation period (after the irradiation procedure was finished). It was found that the residual polymerization processes of nMAG dose gel propagate for up to 6 h after irradiation.

One hour after the irradiation, a gradual decrease of the absorbance values in the irradiated dose gel was identified. A slightly decreasing tendency of the absorbance values was observed up to 6 h after the irradiation. Radiation-induced changes of the optical properties in the post-irradiation phase over the long period might be explained by two competing processes initiated by irradiation: polymerization and the scission of polymers. If low dose rate irradiation is applied, and irradiation time is short, a number of new long chained polymers are created in nMAG dose gel over a certain time period due to the supplied radiation energy. Later, competing polymer scission processes occur that reduce the number of created polymer chains and lead to the setting of the equilibrium between the polymerization and polymer scission processes over the long time period [24]. The contribution of the gelation process to the absorbance changes of irradiated gels should be also considered [21].

**Figure 3:**
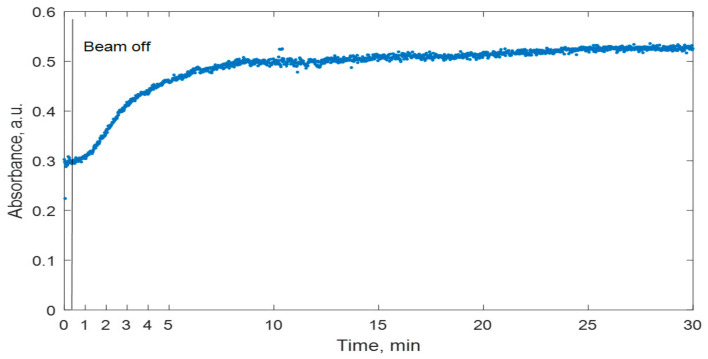
Variations of optical properties in irradiated nMAG dose gel over time period of 30 min.

The performed investigation revealed that the most intensive changes in the UV-Vis spectra of irradiated gels were seen within 30 min after irradiation; therefore, this period was selected for further investigations (Figure 3). It was found that ~95% of the maximum absorbance value recorded by the end of a 30 min interval is achieved during the first 15 min after the start of the irradiation. This correlates well with the initial phase of the most intensive radiation-induced polymerization in dose gels and also indicates that the initiation and propagation of polymerization processes continue significantly longer than the irradiation process itself.

It is important to know what factors could affect the temporal response of the nMAG gel dosimeter. It was reported [25,26] that the influence of irradiation energy on the dose response of methacrylic acid-based dosimeters was negligible [25,26]. On the contrary, the irradiation dose rate contributes significantly to the temporal dose sensitivity of dose gels and is one of the most important parameters for the analysis of polymerization processes in irradiated dose gels over the time after irradiation.

**Figure 4:**
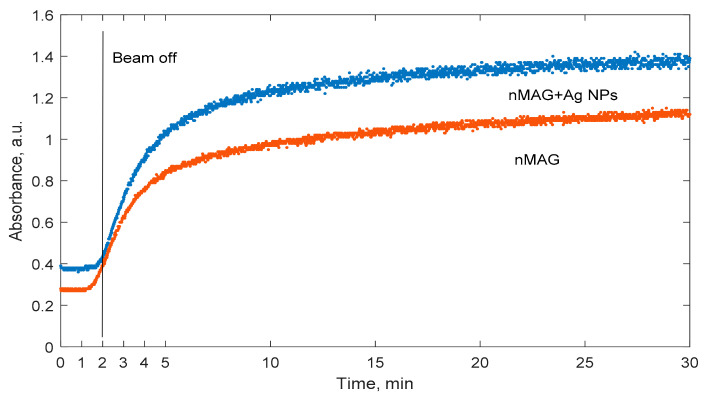
Temporal variations of optical properties of irradiated nMAG gels composed with and without Ag nanoparticles.

The addition of metal nanoparticles is known as a perspective approach to enhance the sensitivity of the gel dosimeter. However, the role of nanoparticles in the polymerization dynamics of dose gels is not clearly defined. Therefore, the polymerization dynamics of nMAG dose gel in the presence of silver nanoparticles was investigated. In order to acquire well-detectable polymerization-induced optical property changes of dose gels within a short (30 min) investigation period, nMAG gels without and with Ag additives (Figure 4) were irradiated up to 4 Gy applying a 200 cGy/min dose rate. The total dose was delivered to the target within 2 min.

It is important to mention that the initial absorbance values of the nMAG dose gel enriched with silver nanoparticles were higher compared to those of the original nMAG gel. At the very beginning of irradiation, the absorbance values of the nMAG gel were increasing faster than those of the nanoparticle-enriched modified gel. This could be connected with the lower polymerization rate due to the lower production of radicals and thus the lower probability for the termination of reactions. However, with time the optical property changes became similar in both gel types. For both types of nMAG gels, 95% of the maximum absorbance value recorded by the end of a 30 min interval was achieved within the first 18.3 min after the start of irradiation. Therefore, it was concluded that the addition of silver nanoparticles to the gel does not affect the time needed for the polymerization process to settle.

**Figure 5:**
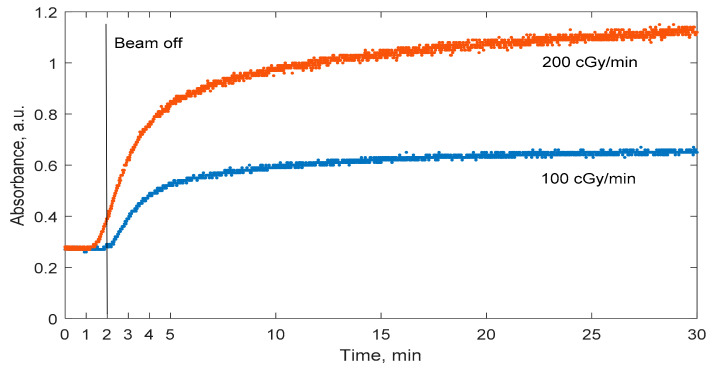
Temporal variations of the optical properties of nMAG gel dosimeter irradiated with different dose rates.

Hannah J. Lee et al. reported that methacrylic acid-based gel dosimeters have a severe dose rate dependence [25]. Since changes in the dose rate affect the extent of polymerization, there is a high possibility that the polymerization dynamics could also be affected. In order to investigate these effects, nMAG dose gel was irradiated with two different dose rates: 100 cGy/min and 200 cGy/min (Figure 5). Irradiation continued for 2 min in both cases.

As expected, dose rate plays an important role in the polymerization dynamics of irradiated nMAG dose gel. The polymerization process settles much faster in gels irradiated with a 100 cGy/min dose rate as compared with a 200 cGy/min dose rate. A 95% change in absorbance was achieved in 18.3 min after the start of the irradiation of the gel when the 200 cGy/min dose rate was used and in 15 min after the start of radiation when the 100 cGy dose rate was used. The dose rate’s impact on the polymerization dynamics is related to the generation of long-term radicals. P. Sathiyaraj et al. [27] concluded that an increased dose rate increases the production of radicals.

**Figure 6:**
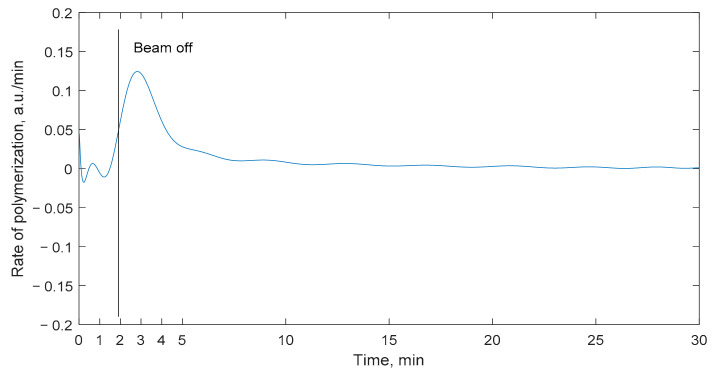
Temporal changes of polymerization rate through time for nMAG gel dosimeter.

In previous investigations, it was shown that the polymerization rate significantly differs throughout the imaging period. For example, during the first minute after the irradiation, the polymerization process develops very slowly. In order to investigate this effect in more detail, the temporal variations of the polymerization rate in the irradiated nMAG dosimeter (dose rate of 100 cGy/min) were investigated (Figure 6). The polymerization reaction rate was considered as a first derivative of the absorbance-time data.

It was found that, during the first minutes after the start of the irradiation, the polymerization process is almost negligible since the amount of created polymer radicals is very low. Fast changes in the absorbance values of the nMAG gels start after 1.5 min, at the near end of the irradiation, when most of the radiolysis reactions are finished and there are plenty of free radicals to react with monomers. Radiolysis reactions develop almost instantly—predominant intermediates take place 10^−8^ s after the irradiation [10]. In the time period from 1.5 to 4 min, the propagation reactions proceed at a higher and higher speed. The reaction speed reaches the maximum value at approximately 4 min after the start of the irradiation. After reaching the maximal polymerization rate, the polymerization process starts to decline in an exponential manner as a consequence of the depletion of radicals and the termination of polymerization reactions.

## 3. Conclusions

A method for polymerization dynamics measurements in irradiated dosimetric gels and a corresponding measurement system were developed.

It was found that changes of the optical properties in irradiated nMAG dosimetric gel can be detected up to 6 h after the start of irradiation. This time is significantly shorter compared to the 24 h recommendation provided by other authors.

It was found that the highest polymerization reaction rate is achieved in 1.5 min after the start of irradiation.

After investigation of the factors affecting polymerization dynamics, it was concluded that the addition of dose sensitivity enhancing silver nanoparticles to the nMAG dosimetric gel did not have a significant influence on the dynamics of polymerization.

The determination of the significantly shorter time interval (compared to 24 h post-exposure recommendations) for dose gel readout and the evaluation of factors contributing to the polymerization dynamics of nMAG dose gels facilitates further investigation of dosimetric gels and their introduction into the medical environment, thus improving the quality of such procedures.

## 4. Materials and Methods

### 4.1. Dosimetric Gels

Based on previous experience and investigations of other researchers, nMAG dosimetric gel was considered as the most suitable candidate for the investigation of irradiated gel’s polymerization dynamics. nMAG gel indicated one of the highest dosimetric sensitivities among gel dosimeters [16]. The nMAG gel dosimeter consists of methacrylic acid, (8% *w*/*w*) which acts as a monomer; gelatin (8% *w*/*w*), which forms a scaffold for the formed polymer network; water as a base of the gel (83.8% *w*/*w*); and tetrakis(hydroxymethyl)phosphonium chloride (THPC) (10 mM), which acts as an oxygen scavenger, because oxygen inhibits the polymerization process. Unlike most polymer gel formulations, nMAG dosimetric gels do not contain a crosslinker, which joins separate polymer chains [28].

The nMAG dosimetric gel was fabricated according to the procedure described by Karlsson et al. [29]. Firstly, gelatin (8% *w*/*w*) was poured into the glass and mixed with distilled water (83.8% *w*/*w*). The glass was heated up to 45 °C until complete dissolution of gelatin. After this, the solution was cooled down to 32 °C and methacrylic acid was added (8% *w*/*w*). The solution was stirred for 25 min with a magnetic stirrer maintaining constant temperature. At the end, THPC was added drop by drop to the gel. The final solution was mixed for 1–2 min. The fabricated gel was poured into standard poly(methyl methacrylate) cuvettes, sealed with laboratory film, and stored for 24 h until irradiation.

The composition of the nMAG gel dosimeter with silver nanoparticles was similar to the original formulation with several modifications. Silver nanoparticles were synthesized from a AgNO_3_ precursor using a trisodium citrate-based chemical synthesis method. The achieved concentration of the nanoparticles was approximately ~6 × 10^27^ particles/mL [30].

The synthesized solution with nanoparticles was exchanged for part of the water in the gel dosimeter. Also, it was noted that the precursor of silver nanoparticles AgNO_3_ tends to react with THPC, forming AgCl precipitates in the gel solution:

AgNO_3_ (aq) + (HOCH_2_)4PCl (aq) → (HOCH_2_)4PNO_3_ (aq) + AgCl (s) (8)

In order to avoid the formation of precipitates, THPC was exchanged with the similar oxygen scavenger tetrakis (hydroxymethyl) phosphonium sulfate (THPS), which does not form insoluble compounds.

In summary, the modified nMAG dose gel enriched with silver nanoparticles consisted of methacrylic acid (8% *w*/*w*), gelatin (8% *w*/*w*), water (53.8% *w*/*w*), solution containing Ag nanoparticles (30% *w*/*w*), and THPS (5 mM). The fabrication procedure of the nMAG gel with Ag nanoparticles was analogous to the original procedure described by Karlsson et al. [29], appending that the solution of silver nanoparticles was added straight away after the addition of methacrylic acid.

**Figure 7:**
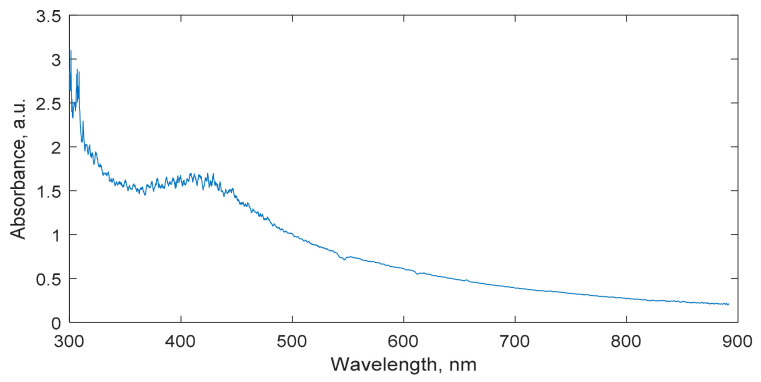
UV-Vis spectrum of nMAG gel dosimeter enriched with Ag nanoparticles with indicated and clearly seen plasmon resonance peak at 410 nm.

The presence of synthesized Ag nanoparticles was characterized using UV-Vis spectrometry. In the acquired UV-Vis spectrum, the plasmonic peak relating to the Ag nanoparticles was clearly visible in the range of 390–420 nm (Figure 7). This indicated that the dominating size of the nanoparticles was between 40 and 50 nm.

### 4.2. Irradiation

The dosimetric gels were irradiated with 6 MeV photon-rays in a linear accelerator Varian Clinac DMX 24 h after fabrication, keeping the following parameters: an SSD of 100 cm and a field size of 10 × 10 cm. The dose rate was chosen depending on the situation: a 300 cGy/min dose rate was used for long-term measurements and 200 cGy/min and 100 cGy/min dose rates were used for the investigation of factors, contributing to temporal dose sensitivity. The selected dose for the investigation of long-term polymerization was 1 Gy, while for short-term investigations it was 4 Gy.

**Figure 8:**
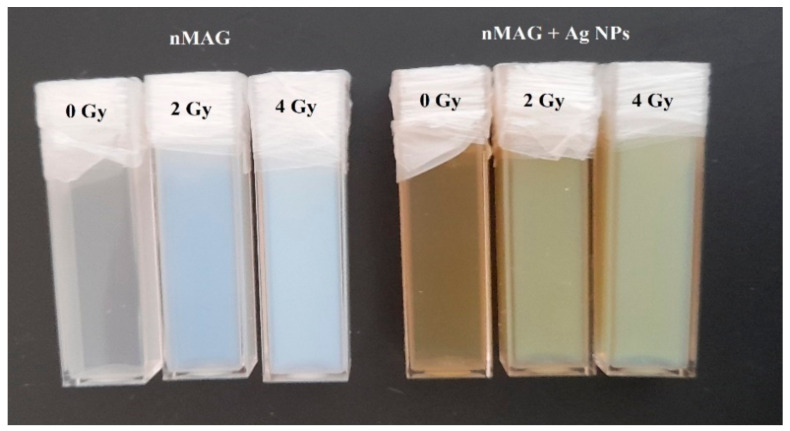
Original and modified with silver nanoparticles nMAG dose gels irradiated to different doses.

An example of irradiated nMAG gels is provided in Figure 8.

### 4.3. Measurement System

Optical interrogation methods are usually used for studying the properties of dosimetric gels. Based on existing experience, an optical measurement system was constructed that allows for real-time studies of the optical properties of dose gels during their irradiation with X-rays and during the post-irradiation phase.

The main optical parameter used for dose evaluation was the optical absorbance. Measurements were performed using a specialized spectrophotometric system, which was modified and adapted to real-time measurements.

**Figure 9:**
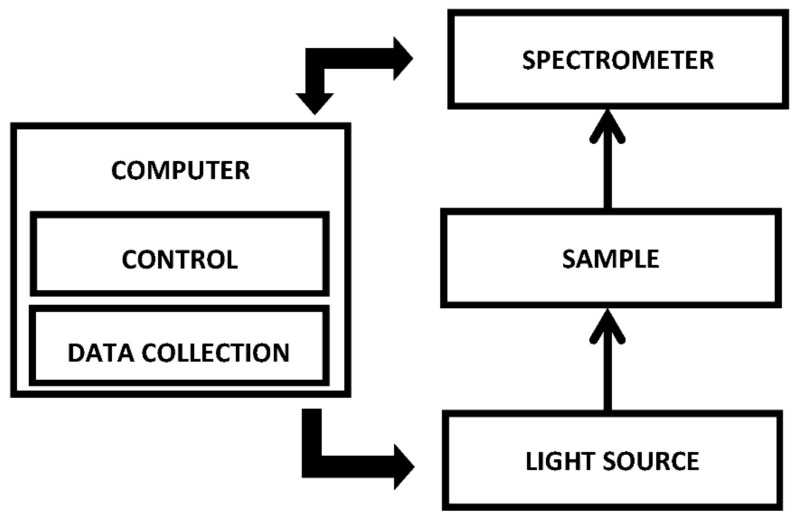
Block diagram of the measurement system.

A block diagram of the designed system is presented in Figure 9.

An Ocean Optics USB4000 spectrometer (200–1000 nm) was used as the main component of the system. The spectrometer was chosen due to its ability to store data at a frequency of up to 10 Hz (depending on the selected data integration time) and due to a possible power supply via the Universal Serial Bus (USB) interface. No additional modifications were required to the spectrometer.

Standard PMMA cuvettes were used for filling with dosimetric gels. A special sample holder was designed and 3D printed, which provided locations for SMA fiber optic connections.

For data acquisition, Ocean Optics Oceanview software with a high mechanical strength tablet Getac T800 was used. The tablet has the ability to connect an additional battery, which is needed for long-term measurements.

Measurements of the optical absorbance spectra of the dose gels in situ were started a few minutes before the irradiation of the dosimetric gels and were performed for relatively long periods of up to 24 h after the irradiation. For this reason, large data sets were obtained with a number of spectra of 10^6^ and a number of data points of 10^8^–10^9^.

Due to the large amount of data, data processing was performed in an automated way using a developed MATLAB algorithm. Data analysis was performed in two stages. In the first stage, the wavelength at which the largest changes of optical properties over time occurred was determined. This wavelength was used for the optimization of data collection and adaption of the measurement system itself to measure a specific wavelength. In the second stage, generation of a graphical representation of the polymerization dynamics for the most sensitive wavelength was implemented.

## Data Availability

The data in this work are available upon request from the corresponding author.
